# Enhancer polymorphism rs10865710 associated with traumatic sepsis is a regulator of PPARG gene expression

**DOI:** 10.1186/s13054-019-2707-z

**Published:** 2019-12-30

**Authors:** Hongxiang Lu, Dalin Wen, Jianhui Sun, Ling Zeng, Juan Du, Dingyuan Du, Lianyang Zhang, Jin Deng, Jianxin Jiang, Anqiang Zhang

**Affiliations:** 1 State Key Laboratory of Trauma, Burns and Combined Injury, Institute of Surgery Research, Daping Hospital, Army Medical University, Changjiang Branch Road 10, Daping Street, Yuzhong District, Chongqing, 400042 China; 2 Department of Cardiothoracic Surgery, Chongqing Emergency Medical Center, The Affiliated Central Hospital of Chongqing University, Chongqing, 400042 China; 3 Department of Emergency Surgery, The Affiliated Hospital of Guizhou Medical University, Guiyang, 550004 Guizhou China

**Keywords:** Peroxisome proliferator-activated receptor gamma, rs10865710, Transcriptional regulation, Trauma, Sepsis

## Abstract

**Background:**

Peroxisome proliferator-activated receptor gamma (PPARγ) is a major regulator in sepsis. Our previous study identified the enhancer polymorphism rs10865710C/G to be associated with susceptibility to sepsis in trauma patients. We performed two-stage cohort studies integrating biological experiments of potential functional variants that modify susceptibility to traumatic sepsis.

**Methods:**

Improved multiplex ligation detection reaction (iMLDR) was used to genotype rs10865710 in 797 Han Chinese trauma patients in Chongqing. Clinical relevance was validated in 334 patients in Guizhou. The potential function of rs10865710 in transcriptional regulation was explored through a dual luciferase reporter assay and electrophoretic mobility shift assay (EMSA). Expression of PPARγ was assessed by expression quantitative trait locus (e-QTL) and western blot analyses.

**Results:**

The association results confirmed rs10865710 to be significantly strongly associated with sepsis risk in trauma patients of the Chongqing and Guizhou cohorts (OR = 1.41 (1.11–1.79), *P* = 0.004 and OR = 1.45 (1.01–2.09), *P* = 0.046, both for allele-dose effect, respectively). A meta-analysis of both cohorts and a previous study indicated strong evidence for this association (OR = 1.41 (1.17–1.71), *P* = 0.0004 for the dominant model, OR = 1.78 (1.34–2.36), *P* < 0.0001 for the recessive model and OR = 1.38 (1.20–1.58), *P* < 0.0001 for the allelic model). Functional experiments verified that rs10865710 was a causative variant influencing enhancer activity (G vs. C, 0.068 ± 0.004 vs. 0.096 ± 0.002, *P* = 0.0005) and CREB2 binding. Expression analysis also indicatevd rs10865710 genotypes to be associated with levels of PPARγ expression (*P* = 9.2 × 10^−5^ for dominant effect and *P* = 0.005 for recessive effect).

**Conclusions:**

Our study provides evidence that the enhancer-region polymorphism rs10865710 might influence transcription factor binding and regulate PPARγ expression, thus conferring susceptibility to traumatic sepsis.

**Trial registration:**

ClinicalTrials.gov, NCT01713205. Registered 18 October 2012, retrospectively registered.

## Introduction

Trauma is one of the leading causes of death worldwide. One of the most serious complications following major trauma is sepsis and sequential dysfunction of vital organs [1]. Individuals differ greatly in their response to infection, likely due to external (e.g., the site of infection and virulence and type of pathogenic bacteria) and host (e.g., sex, age, concomitant disease, and resistance) factors. Importantly, host genetic factors are also recognized as having a great impact on the occurrence and progression of sepsis [2]. In general, knowledge of genetic factors will allow for early identification of high-risk trauma patient populations with sepsis, facilitating the prompt initiation of anti-infection therapy and treatment to support organ function and thereby improving prognosis.

A tremendous amount of inflammatory mediator production is an important feature of traumatic sepsis [3]. Peroxisome proliferator-activated receptor gamma (PPARγ) is a ligand-dependent transcription factor of the nuclear receptor superfamily with several biological functions, including control of the inflammatory response [4]. PPARγ is widely expressed in various immune cells, such as neutrophils, lymphocytes, monocytes/macrophages, and dendritic cells [5]. Previous studies have indicated that PPARγ exerts potent anti-inflammatory effects by modulating neutrophil migration and activation; enhancing macrophage phagocytosis; regulating inflammatory mediator production such as tumor necrosis factor-α (TNF-α), interleukin-1β (IL-1β), IL-6, IL-8, and IL-10; and activating oxygen/nitrogen species [6]. For example, the level of PPARγ expression was found to be reduced in freshly isolated mononuclear cells from sepsis patients [7] as well as in the lungs in sepsis murine models [8]. Numerous in vivo and in vitro studies have shown that administration of PPARγ agonists can suppress the inflammatory responses associated with sepsis [9], acute lung injury [10], and inflammation [11], increasing the host’s ability to kill and clear pathogenic bacteria and improving prognosis. Accordingly, PPARγ and its ligands have become novel therapeutic targets for the treatment of sepsis and other inflammatory diseases. Although PPARγ is consistently annotated as a potent anti-inflammatory molecule, the functional variants and the underlying mechanisms involved have not yet been identified.

Patients carrying a mutated PPARG gene might be susceptible to excessive inflammatory responses, sepsis, and/or poor prognosis. Our previous pilot study [12] revealed strong clinical relevance for rs10865710C/G, with a higher incidence of sepsis and multiple organ dysfunction (MOD) scores with the variant G allele in trauma patients. Further analysis indicated that rs10865710G was significantly associated with high levels of the pro-inflammatory cytokine TNF-α in the plasma of trauma patients in response to lipopolysaccharide (LPS) stimulation. Given the central role of PPARG in the pathogenesis of traumatic sepsis, we hypothesize that rs10865710 polymorphism may impact PPARG expression and the occurrence of sepsis in trauma patients. Thus, we evaluated two additional populations to further confirm the clinical association of rs10865710 with the risk of developing sepsis in trauma patients. Additionally, we explored whether there is a functional link between rs10865710 and PPARG transcription and protein expression. Such a mechanistic characterization of this sepsis-associated functional polymorphism might provide new opportunities for the development of targeted therapy for traumatic sepsis.

## Methods

### Study populations

Two unrelated cohorts of major trauma patients from Chongqing (internal test cohort) and Guizhou (external validation cohort) in China were assessed in this study. Major trauma patients were consecutively recruited if they met previously described criteria [13] from the department of trauma surgery or intensive care unit (ICU) at Daping Hospital and Chongqing Emergency Medical Center (Chongqing) from 2014 to 2016 and Department of Trauma and Emergency at the Affiliated Hospital of Guizhou Medical University (Guizhou) between 2010 and 2013. Briefly, the inclusion criteria were as follows: (1) the age of trauma patients is between 18 and 65 years, (2) patients with Injury Severity Score (ISS) more than 16, and (3) patients surviving more than 48 h after injury. Patients were not eligible if they had penetrating injuries, severe brain injury (Abbreviated Injury Scale of the head ≥ 3), preexisting organ dysfunction, or immune diseases. Whole blood specimens were collected within 2 h of admission to the hospital. Ethics approval for this study was obtained from the Ethical and Protocol Review Committees of Army Medical University, Chongqing Emergency Medical Center, and Guizhou Medical University. Informed written consent was obtained from the patients and their next of kin before enrollment, including explicit permission for DNA analysis and the collection of relevant clinical data. Patient confidentiality was preserved according to the guidelines of the Declaration of Helsinki.

### Clinical evaluation

All patients requiring surgical intervention received standard surgical care and postoperative ICU treatment. The Injury Severity Score (ISS) was calculated according to the Abbreviated Injury Scale developed in 2005. Sepsis was diagnosed for trauma patients who had a rapid change of SOFA score ≥ 2 with confirmed or suspected infection during the hospital period according to “Sepsis-3.0” [14]. Infection was defined as a clinically obvious source or positive bacterial culture. Daily physiologic and laboratory data were obtained during the hospital stay, and clinical events were recorded thereafter until death or discharge from the hospital. MOD scores were calculated as the sum of the simultaneously obtained individual organ scores on each hospital day [15]. MOD scores and the presence of sepsis were determined by individuals who were unaware of the genotyping results.

### Genotyping

Genomic DNA was extracted from whole blood using a Wizard genomic DNA purification kit (Promega, USA), and rs10865710 genotyping was performed using the improved multiplex ligation detection reaction (iMLDR) technique (Genesky Biotechnologies Inc., China), as described by our previous study [12]. Genotyping was performed in a blinded fashion without knowledge of the patients’ clinical data, and 10% of randomly selected samples were further confirmed by direct sequencing.

### Bioinformatics analysis

As rs10865710 is located in the intron region of the PPARG gene and previous evidences [16] have suggested that intron might function as enhancer, we speculated that rs10865710 may affect enhancer activity and further influence expression of PPARG. We used the database Ensembl (http://aisa.ensembl.org/index.html) to search for any enhancer segment in the rs10865710 region and then used TFSEARCH (http://www.cbrc.jp/research/db/TFSEARCH.html) to predict putative transcription factor binding sites and perform validation. Furthermore, we conducted expression quantitative trait locus (e-QTL) analysis using data from the NCBI PheGenI database (https://www.ncbi.nlm.nih.gov/gap/phegeni).

### PPARɣ protein expression

The protein levels of PPARɣ from trauma patients were detected by western blotting according to a standard protocol. Briefly, white cells were lysed in RIPA buffer, and the protein concentration of the supernatant was quantified using BCA Protein Assay Kit (Beyotime, China). Proteins were separated by sodium dodecyl sulfate-polyacrylamide gel electrophoresis (SDS-PAGE) and transferred to nitrocellulose (NC) membranes (Millipore, USA) membrane. Following incubation for 1 h with 5% nonfat milk at room temperature, the membranes were incubated with a specific primary antibody against PPARɣ (Cell Signaling Technology, USA) overnight at 4 °C. The membranes were then incubated with the secondary antibody for 1 h at room temperature. Signals were detected by an Odyssey infrared scanner (LI-COR Biosciences, USA). The intensity of the bands was determined by densitography, and β-actin was used as a loading control.

### Enhancer activity assays

The transcriptional regulation of rs10865710 in the enhancer region of the PPARG gene was revealed by a reporter gene assay system [17]. As previously reported [13], a 1000-bp regulatory region of the PPARG enhancer with the rs10865710 C or G allele was inserted into the pGL3-promotervector (Promega, USA) to construct two different plasmids. The reporter constructs were introduced into HEK293T cells using an NEPA21 electroporator at a 125-V poring pulse voltage and 5.0-ms poring time (Nepa Gene, Japan). A total of 5 × 10^5^ cells were resuspended in 100 μl electroporation solution and mixed with 10 μg reporter plasmid DNA and 200 ng internal control plasmid (pRL-CMV). Transiently transfected cells were grown for 48 h before assaying with a Dual-Luciferase Reporter Assay System (Promega, USA) and Luminoskan Ascent luminometer (Thermo Labsystems, Finland). Relative luciferase activity was calculated as the ratio of luminescence for the experimental reporter to that of the internal control plasmid (pRL-CMV). Statistical significance was calculated using the two-tailed *t* test with three biological replicates.

### Electrophoretic mobility shift and super-shift assays

Nucleoprotein was extracted from the mononuclear cell line THP-1 using the NE-PER nuclear and cytoplasmic extraction kit (ThermoFisher Scientific, USA) according to the manufacturer’s instructions. Infrared dye DY-682-labeled (Eurofins Genomics, Germany) and unlabeled complementary oligonucleotides flanking rs10865710 were annealed to generate a double-stranded EMSA probe (5′TTGGCATTAGATGCTGTTTTGTCTT[C/G]ATGGAAAATACAGCTATTCTAGGAT3′-biotin). Nuclear protein was incubated with the labeled probes, and DNA-protein complexes were resolved by electrophoresis on a 6% polyacrylamidegel (Life Technologies, USA). Gels were imaged using Odyssey Fc Infrared Imaging System (LI-COR Biosciences, USA). For competition experiments, 10, 200-fold excess unlabeled competitor probe was pre-incubated with the nuclear extract before biotin-labeled probes were added. For the super-shift assay, 2 μg or 4 μg of rabbit anti-human CREB2 monoclonal antibody (Abcam, UK) was added to the binding reaction. Biotin-labeled DNA-protein complexes were detected by chemiluminescence.

### Statistical analysis

Continuous data are presented as the mean ± standard deviation (SD). Categorical data are presented as counts and percentages. Hardy-Weinberg equilibrium (HWE) was tested using *χ*^2^ analyses. Three different genetic models, including dominant, recessive, and allele-dose models, were applied to analyze the relevance of rs10865710C/G for clinical outcomes. Multivariable logistic regression models were used to calculate odds ratios (OR) with 95% confidence intervals (CIs) to assess sepsis risk, adjusting for age, sex, and injury severity for confounding effects. The association of the polymorphism with MOD scores was determined by linear regression analysis, adjusting by age, sex, and injury severity for confounding effects. Meta-analysis of the combined two validated cohorts and our previous study was performed using the Cochran-Mantel-Haenszel statistical test, and heterogeneity was assessed by calculating *I*^2^, as described by Higgins et al. [18]. For each comparison, the exact *P* values, considered significant at < 0.05, are reported. Data were analyzed using SPSS 11.5 statistical software (SPSS Inc., USA).

## Results

### Characteristics of the study population

Patients in the internal test (Chongqing, *n* = 797) and external validation (Guizhou, *n* = 334) cohorts were predominantly male and middle aged (Additional file 2: Table S1). The internal test and external cohorts were newly enrolled ethnic Han Chinese individuals, similar to our previous pilot study cohort [12]. The patients had sustained severe injuries (mean ISS 22.6 ± 7.9 and 23.4 ± 8.2). The median periods from injury to admission to hospital were 12 h (1–48 h) and 16 h (1–48 h) in the two cohorts, respectively. Sepsis morbidity rates were 34.9% and 37.1% in the two cohorts, respectively. Median time point for sepsis occurrence was 6 days (interquartile range 4.5–8.5 days) and 5 days (interquartile range 4.0–7.0 days). Organ dysfunction occurred in 34.4% and 40.4%, respectively, among whom 87 (31.8%) and 47 (34.8%) exhibited dysfunction in more than two organs. The median time point for MODS occurrence was found to be 7 days (interquartile range 5.5–10.0 days) and 6 days (interquartile range 4.5–8.5 days), respectively.

### Associations of rs10865710 in the internal test trauma cohort

First, we selected successful genotyping 797 trauma patients from the Chongqing district to investigate associations of rs10865710 (Additional file 1: Fig. S1). The minor allele frequency (MAF) of rs10865710 was 33.9%, and the genotype distribution was consistent with HWE (*P* = 0.73, Additional file 3: Table S2). There were no significant differences in sex ratio, age, and ISS among the patients when stratified according to the different genotypes of rs10865710. As expected, trauma patients carrying the rs10865710G allele had a significantly increased risk of sepsis and higher MOD scores than those carrying the C allele (odds ratio (OR) = 1.44, 95% confidence interval (CI) = 1.07–1.94, *P* = 0.016 for sepsis risk and *P* = 0.005 for MOD scores in the case of a dominant effect). Data from multiple regression analyses further indicated that this association had a significant allele-dose effect on the sepsis morbidity rate (OR = 1.41, 95% CI = 1.11–1.79, *P* = 0.004) and MOD score (*P* = 0.002) (Table 1).

**Table 1:** Clinical relevance of the rs10865710C/G in patients with major trauma

Cohorts	Genotype	*N*	Age years	Gender M/F (%)	ISS	Sepsis, *n* (%)	MOD score
Internal test	CC	350	43.1 ± 13.3	278 (79.4%)/72 (20.6%)	22.4 ± 7.6	106 (30.3)	4.8 ± 2.1
	CG	353	43.0 ± 12.3	284 (80.5%)/69 (19.5%)	22.5 ± 8.3	130 (36.8)	5.7 ± 2.2
	GG	94	42.0 ± 12.4	83 (88.3%)/11 (11.7%)	23.7 ± 8.0	42 (44.7)	6.0 ± 2.6
						a1, b1, c1	a2, d1
External validation	CC	144	38.4 ± 12.6	112 (77.8%)/32 (22.2%)	23.3 ± 9.9	44 (30.6)	4.1 ± 3.5
	CG	153	38.1 ± 12.4	129 (84.3%)/24 (15.7%)	24.8 ± 8.7	63 (41.2)	4.9 ± 3.2
	GG	37	40.5 ± 11.7	31 (83.8%)/6 (16.2%)	23.0 ± 8.4	17 (45.9)	6.2 ± 2.9
						a3, c2	a4, b2, d2

Age, ISS, and cytokine are given as mean ± standard deviation; MOD score is given as mean ± standard error

*F* female, *ISS* Injury Severity Score, *M* male, MOD multiple organ dysfunction

a: Dominant effect (GG + CG versus CC) as analyzed by analysis of covariance, ^a1^*P* = 0.016, ^a2^*P* = 0.0005, ^a3^*P* = 0.03, ^a4^*P* = 0.009

b: Recessive effect (GG versus CG + CC) as analyzed by analysis of covariance, ^b1^*P* = 0.034, ^b2^*P* = 0.024

c: The relative risk of sepsis as analyzed by logistic regression analyses, adjusting for age, sex, and injury severity for confounding effects, ^c1^*P* = 0.004, OR = 1.41, 95% CI = 1.11–1.79; ^c2^*P* = 0.046, OR = 1.45, 95% CI = 1.01–2.09

d: The relative risk of MOD score as analyzed by linear regression analyses, adjusting for age, sex, and injury severity for confounding effects, ^d1^*P* = 0.002, ^d2^*P* = 0.002

### Validation of associations for rs10865710 in Guizhou trauma patients

We further confirmed the results in an additional trauma cohort (Guizhou). Due to the similar locations of the two cohorts (both in the southwestern region of China), clinical characteristics and genotype distribution were similar (Additional file 2: Table S1 and Additional file 3: Table S2). As shown in Table 1, we found similar associations with the risk of posttraumatic sepsis in the validation cohort. The sepsis morbidity rate was significantly higher in patients carrying the variant G allele than the wildtype C allele (OR = 1.65, 95% CI = 1.05–2.61, *P* = 0.03 for sepsis incidence and *P* = 0.009 for MOD score in the dominant model). Data from multiple regression analyses also showed that the association of this polymorphism with a higher risk of sepsis (OR = 1.45, 95% CI = 1.01–2.09, *P* = 0.046) and a higher MOD score (*P* = 0.002) in Guizhou trauma patients was significant.

### Results of meta-analysis

Meta-analysis of the results from the Chongqing and Guizhou cohorts and our previous study was performed for rs10865710 in a total of 1865 trauma patients (Table 2). For traumatic sepsis, no obvious evidence of heterogeneity was observed using any of the three genetic models (*I*^2^ = 0). Thus, we selected a fixed-effects model to pool the combined OR. In the dominant model (GG vs. CG + CC), the overall pooled OR for the three studies combined was 1.41 (95% CI = 1.17–1.71) (*P* = 0.0004). Similarly, the recessive and allelic models demonstrated significant associations with sepsis risk (recessive model OR = 1.78, 95% CI = 1.34–2.36, *P* < 0.0001; allelic model OR = 1.38, 95% CI = 1.20–1.58, *P* < 0.0001). Regarding the MOD score, the meta-analysis also supported the association of rs10865710 in both dominant (MD = 0.70 score, 95% CI = 0.09–1.31, *P* = 0.02) and recessive (MD = 0.91 score, 95% CI = 0.56–1.27, *P* < 0.0001) models.

**Table 2:** Meta-analysis of rs10865710 association with sepsis and organ dysfunction in trauma patients

		Sepsis/non-sepsis			MOD score		
Author	*N*	CC	CG	GG	CC	CG	GG
Internal test	797	106/244	130/223	42/52	4.8 ± 2.1	5.7 ± 2.2	6.0 ± 2.6
External validation	334	44/100	63/90	17/20	4.1 ± 3.5	4.9 ± 3.2	6.2 ± 2.9
Gao 2016*	734	123/193	136/192	53/37	4.8 ± 2.2	4.8 ± 1.9	5.6 ± 2.4
Meta-analysis	1865	a1, b1, c1			a2, b2		

*Represented our previous study

a: Dominant effect (GG + CG versus CC) as analyzed by analysis of covariance, ^a1^*P* = 0.0004, OR = 1.41, 95% CI = 1.17–1.71, *I*^2^ = 0, *P*_*h*et_ = 0.67; ^a2^*P* = 0.02, MD = 0.70 score, 95% CI = 0.09–1.31, *I*^2^ = 86%, *P*_het_ = 0.001

b: Recessive effect (GG versus CG + CC) as analyzed by analysis of covariance, ^b1^*P* < 0.0001, OR = 1.78, 95% CI = 1.34–2.36, *I*^2^ = 0, *P*_het_ = 0.59; ^a2^*P* < 0.0001, MD = 0.91 score, 95% CI = 0.56–1.27, *I*^2^ = 26%, *P*_het_ = 0.26

c: Allelic effect (G versus C) as analyzed by analysis of covariance, ^c1^*P* < 0.0001, OR = 1.38, 95% CI = 1.20–1.58, *I*^2^ = 0, *P*_het_ = 0.97

### Results of bioinformatics analysis

We used the database Ensembl to search the fragment with potential enhancer function containing rs10865710, and the fragment chr3:12311574-12312573 showed enhancer activity. We analyzed expression data from the NCBI PheGenI database and found that rs10865710 genotype was significantly associated with PPARG mRNA expression (*P*_dominant_ = 1.2 × 10^−3^), with individuals carrying the CG (6.58 ± 0.15) or GG (6.20 ± 0.12) genotype having a lower level of PPARG in human peripheral leukocytes than individuals carrying the CC (6.82 ± 0.16) genotype.

### Association of rs10865710 with PPARG expression

**Fig. 1:**
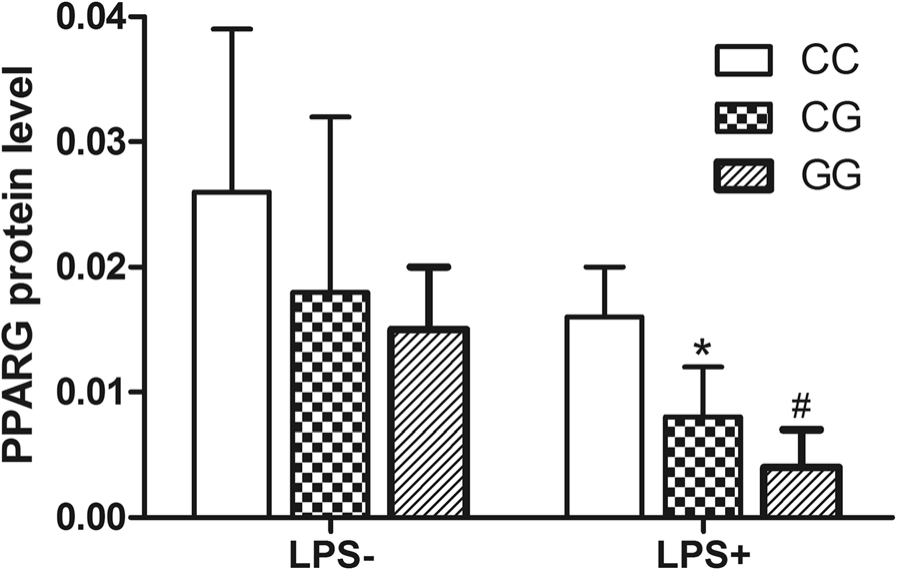
Rs10865710 is associated with lower LPS-induced PPARG protein expression. Whole blood samples were collected from 30 trauma patients (CC: *n* = 10, CG: *n* = 10, GG: *n* = 10, respectively). PPARG expression by peripheral leukocytes was detected using western blotting and is presented as a gray value. *P = 9.2 × 10^−5^ for a dominant association (CG + GG vs. CC), ^#^*P* = 0.005 for a recessive association (GG vs. CG + CC). Student’s *t* test was used to assess statistical significance

To explore the link between rs10865710 and PPARG expression, the protein level of PPARγ was detected in leukocytes exposed ex vivo to LPS stimulation, which were random selected from trauma patients with different genotype in Chongqing district, respectively (CC: *n* = 10, CG: *n* = 10, GG: *n* = 10). The results well associated rs10865710 of different genotypes, showing a significant difference in the case of dominant (*P* = 9.2 × 10^−5^) and recessive (*P* = 0.005) effects (genotype relative gray ratio: CC, 0.026 ± 0.013; CG, 0.018 ± 0.014; GG, 0.015 ± 0.006; Fig. 1).

### Influence of rs10865710 on transcriptional regulation of PPARG mRNA

**Fig. 2:**
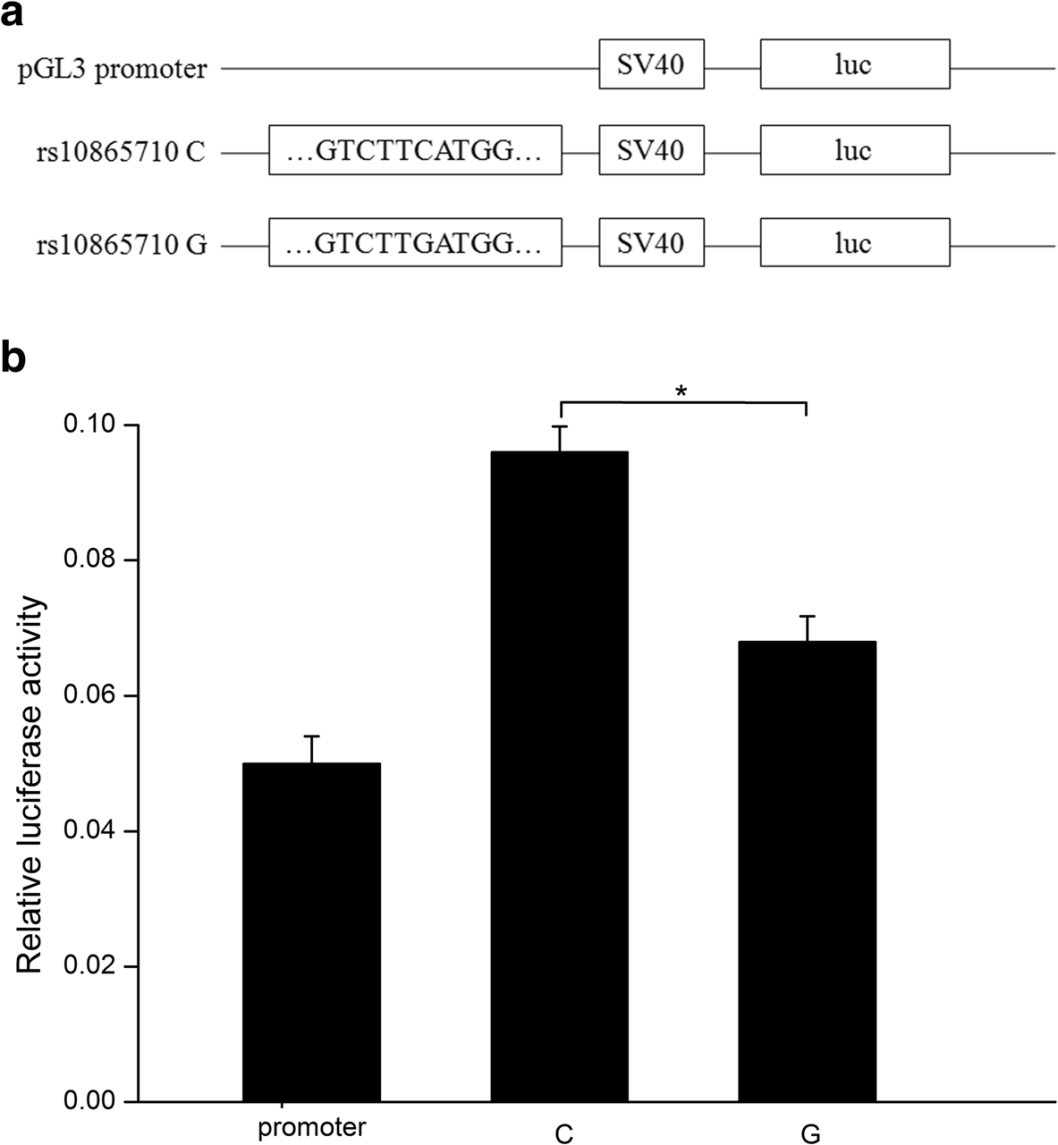
Rs10865710 decreased PPARG transcriptional enhancer activity. **a** Plasmid constructs used for transfection. **b** Transcriptional enhancer activities of rs10865710 measured by luciferase (luc) activity 48 h after transfection. Values of relative luciferase activity are shown as means ± SDs from three independent experiments. *P = 0.005, Student *t* test. C, C allele; G, G allele

We next conducted functional analysis of the rs10865710 polymorphism. Because rs10865710 is located in intron 1 of PPARG, we examined whether the genomic region around rs10865710 has enhancer activity, as predicted through bioinformatics analysis. First, we generated plasmids and conducted luciferase assays to examine whether allelic differences alter the efficiency of PPARG transcription in HEK293T cells. The allele-specific constructs containing the sepsis risk allele (G) ofrs10865710 showed lower transcriptional enhancer activity than did the other construct containing the C allele ofrs10865710 (Figs. 2, 0.068 ± 0.002 vs. 0.096 ± 0.002, *P* = 0.0005). These experiments were performed three times with similar results. Overall, the results demonstrate that rs10865710 variants significantly influence enhancer activity and that the risk allele rs10865710G has lower enhancer activity compared to the non-risk allele rs10865710C.

### Identification of CREB-2 as the transacting factor binding to the PPARG enhancer region

**Fig. 3:**
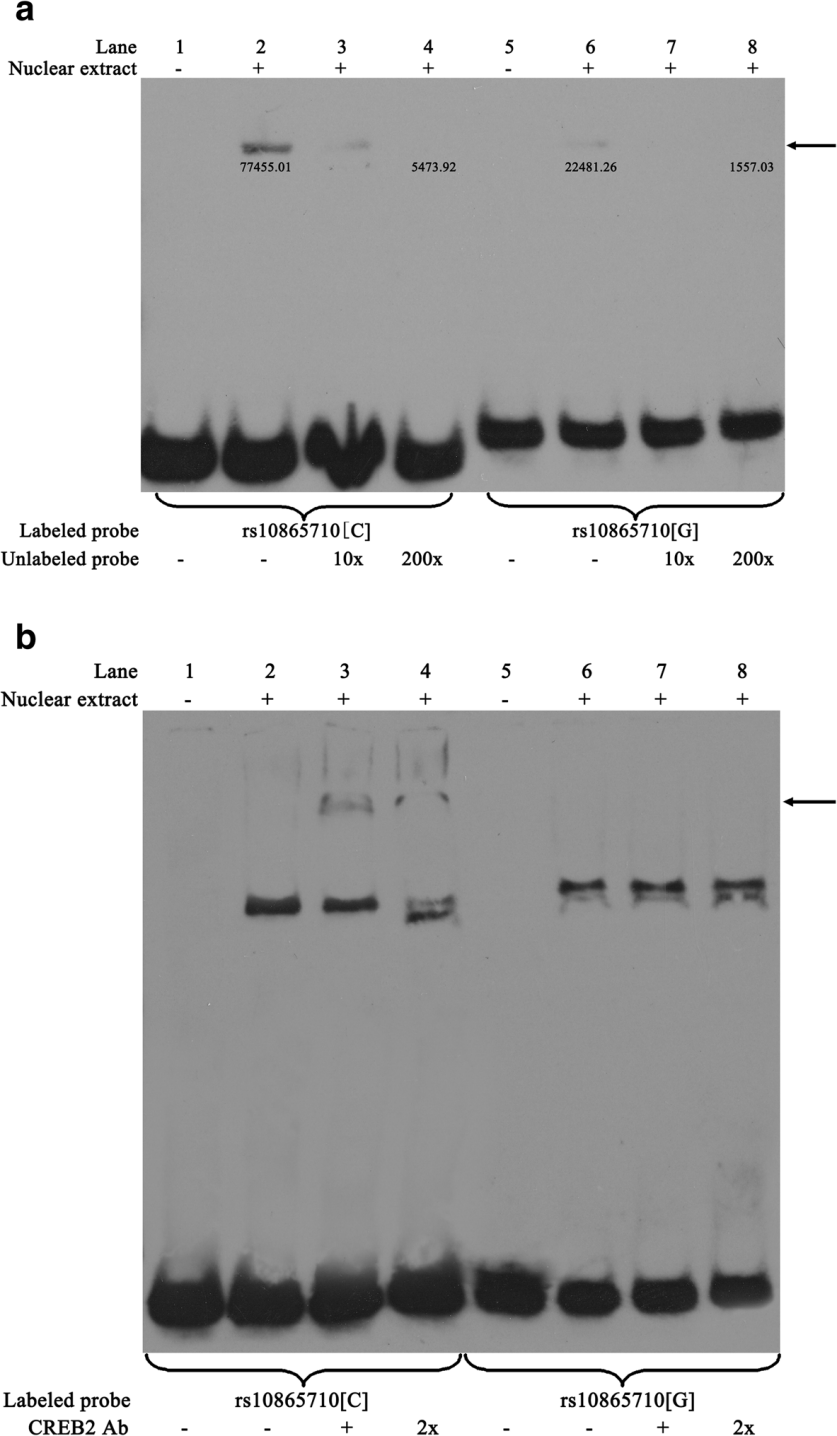
The rs10865710 risk allele disrupts transcription factor CREB2 binding. **a** EMSA with biotin-labeled probes containing either the C or the G allele of rs10865710, incubated with THP-1 cell nuclear extract. The arrow indicates an allele-specific band that interacts with nuclear protein. 10× and 200× indicate 10- and 200-fold unlabeled probes excess over labeled probes. “+” and “−” mean added and unadded, respectively. **b** Super-shift EMSA of rs10865710. Two independent experiments were performed with similar results. Ab, antibody; C, C allele; G, G allele

As rs10865710C/G may play a role in traumatic sepsis susceptibility and transcriptional regulation of PPARG mRNA, we next focused on determining which transacting factor binds to rs10865710C/G. In silico searches using TFSEARCH revealed that the genomic region containing the rs10865710 G allele has lost a consensus sequence corresponding to the putative binding element of CREB2. We examined allelic differences with regard to the binding of nuclear proteins using EMSA. As shown in Fig. 3a (arrow), the signal intensity of the DNA-protein complex derived with the G allele was lower than that with the C allele in the presence of THP-1 nuclear extract, suggesting differential transcription factor binding dependent on the rs10865710 allele. Self-competition with a 200-fold excess of unlabeled “C”-allele probe led to complete abrogation of this band (lane 4). Furthermore, the addition of an anti-CREB2 antibody resulted in a super-shift with the rs10865710C-allele probe (lanes 3 and 4), which indicates that CREB2 is a component of this complex (Fig. 3b).

## Discussion

Our current two-stage cohort genetic association studies integrating biological experiments confirmed that the G allele of rs10865710 was significantly associated with an increased risk of traumatic sepsis, and different alleles (C/G) of rs10865710 might affect enhancer activity through differential transcription factor binding, as revealed by dual luciferase reporter assays and EMSAs. Therefore, integrating the results of e-QTL analysis and the important role that rs10865710 plays in regulating expression of PPARG, we propose that rs10865710 contributes to traumatic sepsis susceptibility by impacting transcription and further affecting expression of PPARG.

PPARG is one of the three subtypes of PPARs and is by far the most widely studied. PPARG forms heterodimers with retinoid X receptors (RXRs), influencing transcription of many target genes. The PPARG gene is located at chromosome3 p25 and contains eight exons; the expressed protein contains 479 amino acids. A common polymorphism (rs10865710) in PPARG is a C → G substitution in intron 1. The MAF of this polymorphism is similar between different populations (fluctuating between 21 and 36%) according to the 1000 Genomes Project. In our study, the frequency of the G allele was approximately 34%, similar to that of the CHB population reported by the 1000 Genomes Project. Moreover, previous studies have suggested a potential correlation of this SNP with systemic sclerosis [19], obesity [20], asthma [21, 22], coronary artery disease [23], and lipid metabolism [24, 25], with most suggesting that variation in rs10865710 is a disease risk factor. In addition, our previous study [12] analyzing correlation between PPAR polymorphisms and sepsis risk found that rs10865710G allele carriers had higher sepsis morbidity, MOD scores, and LPS-induced TNF-α production, which was confirmed in the present study. Functional studies have shown that the C → G substitution results in a decreased binding capacity of transcription factor CREB2 to the enhancer region of PPARG, reducing transcriptional activity, as shown by dual luciferase reporter assays and EMSAs. Furthermore, the results of e-QTL and western blotting revealed that individuals carrying rs10865710CG/GG genotypes express lower levels of PPARγ than do individuals carrying the CC genotype, which indicates that the C to G transition may downregulate PPARγ expression, which may contribute to increase TNF-α production by peripheral blood leukocytes. One previous study [8] reported that PPARγ exerts anti-inflammatory effects through transrepression of the inflammatory activities of nuclear factor κB (NF-κB), signal transducers and activators of transcription 1 (STAT-1), and activator protein-1 (AP-1) via physical interaction, thereby influencing the occurrence and progression of sepsis.

To the best of our knowledge, this is the first study to comprehensively explore the potential regulation of thers10865710 polymorphism in traumatic sepsis. Nevertheless, several limitations of our study should be acknowledged. First, our clinical study was restricted to Han Chinese patients, and whether the findings can be generalized to other ethnic groups needs further evaluation. Second, the relatively small sample size of the second stage resulted in limited statistical power (68% at a significance of 0.05). Additional large studies are needed for validating the clinical relevance of this polymorphism. Finally, as we were unable to obtain additional blood samples to determine PPARG mRNA levels, we could only make our inference based on e-QTL data from the present database, which was further confirmed through protein expression analysis.

## Conclusion

Our two-stage cohort genetic association study integrating biological experiments provided solid evidence that rs10865710 may decrease transcriptional activity and PPARG expression, thus conferring susceptibility to traumatic sepsis. Our findings provide additional insight into rs10865710 as a novel predictive biomarker to improve the early identification of high risk for traumatic sepsis or MODS. Further studies with larger sample sizes and indifferent ethnic populations are warranted to validate the findings, and more biological experiments are also needed to explore the role of rs10865710 in the pathogenesis of traumatic sepsis.

## Supplementary information

**Additional file 1: Figure S1.** The flow chart of trauma sample screening for genetic association and expression analysis of rs10865710.

**Additional file 2: Table S1.** Overall characteristics of patients with major trauma.

**Additional file 3: Table S2.** Distribution of the rs10865710C/G in the PPARG among trauma patients.

## Data Availability

The datasets used for analysis during the current study are available from the corresponding author on reasonable request.

## Abbreviations

AP-1: Activatorprotein-1
CI: Confidence interval
CREB: cAMP-response element binding protein
DNA: Deoxyribonucleic acid
EMSA: Electrophoretic mobility shift assay
e-QTL: Expression quantitative trait locus
HWE: Hardy-Weinberg equilibrium
ICU: Intensive care unit
IL: Interleukin
iMLDR: Improved multiplex ligation detection reaction
ISS: Injury Severity Score
LPS: Lipopolysaccharide
MAF: Minor allele frequency
MOD: Multiple organ dysfunction
NC: Nitrocellulose
NF-κB: Nuclear factor κB
OR: Odds ratios
PPARγ: Peroxisome proliferator-activated receptor gamma
RXR: Retinoid X receptor
SD: Standard deviation
SDS-PAGE: Sodium dodecyl sulfate-polyacrylamide gel electrophoresis
SNP: Single nucleotide polymorphism
SOFA: Sepsis-related Organ Failure Assessment
STAT-1: Signal transducers and activators of transcription 1
TNF: Tumor necrosis factor
