# Supplementary figures and images for: Enhancer polymorphism rs10865710 associated with traumatic sepsis is a regulator of PPARG gene expression
Source: Crit Care. 2019 Dec 30;23:430. doi: 10.1186/s13054-019-2707-z (PMC6938012; doi:10.1186/s13054-019-2707-z)

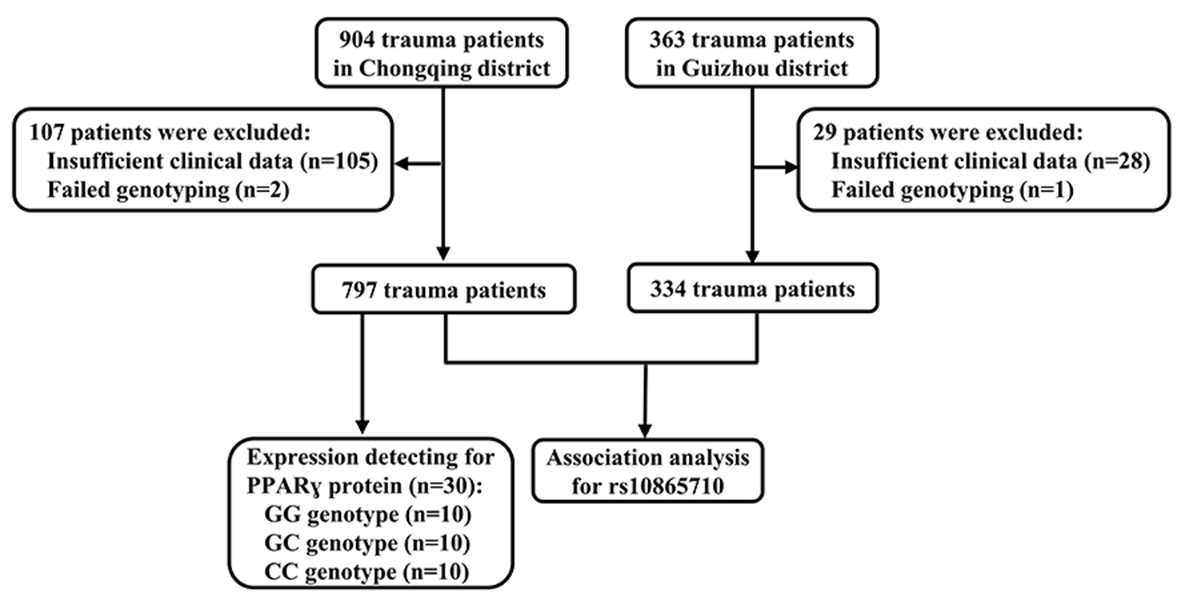

Supplement: Supplementary file 1 — Additional file 1: Figure S1. The flow chart of trauma sample screening for genetic association and expression analysis of rs10865710. [file 13054_2019_2707_MOESM1_ESM.tif]
