# Supplementary material for: Enhancer polymorphism rs10865710 associated with traumatic sepsis is a regulator of PPARG gene expression
Source: Crit Care. 2019 Dec 30;23:430. doi: 10.1186/s13054-019-2707-z (PMC6938012; doi:10.1186/s13054-019-2707-z)
Supplement: Supplementary file 2 — Additional file 2: Table S1. Overall characteristics of patients with major trauma. [file 13054_2019_2707_MOESM2_ESM.docx]

**Table S1. Overall characteristics of patients with major trauma.**

| **Characteristics** | **Internal Test (n=797)** | **External Validation (n=334)** |
| --- | --- | --- |
| Age (yrs) | 42.9±12.8 | 40.5±12.4 |
| Male/female, n | 645/152 | 272/62 |
| Injured body regions, n (%) |  |  |
| Head | 308 (38.6) | 175 (52.4) |
| Thorax | 465 (58.3) | 168 (50.3) |
| Abdomen | 257 (32.2) | 107 (32.0) |
| Extremities | 562 (70.5) | 197 (59.0) |
| Number of regions injured, n (%) |  |  |
| Two | 525 (65.9) | 257 (76.9) |
| Three | 201 (25.2) | 60 (18.0) |
| All four | 71 (8.9) | 16 (4.8) |
| ISS | 22.6±7.9 | 23.4±8.2 |
| ≥16, <25, n (%) | 587 (73.7) | 223 (66.8) |
| ≥25, n (%) | 210 (26.3) | 111 (33.2) |
| Organ dysfunction, n (%) | 274 (34.4) | 135 (40.4) |
| One | 187 (23.5) | 88 (26.3) |
| Two | 55 (6.9) | 32 (9.6) |
| Three or above | 32 (4.0) | 15 (4.5) |
| Sepsis, n (%) | 278 (34.9) | 124 (37.1) |
| Source of infection, % |  |  |
| Respiratory tract infection | 43.5 | 42.3 |
| Primary bloodstream infection | 19.5 | 21.7 |
| Urinary tract infection | 18.5 | 17.6 |
| Catheter associated infection | 9.2 | 9.1 |
| Wound infection | 7.1 | 7.5 |
| Others* | 2.2 | 1.8 |
| Pathogens, % |  |  |
| Gram-negative | 33.8 | 8.1 |
| Gram-positive | 8.6 | 8.9 |
| Fungi | 6.1 | 1.6 |
| Mixed Gram negative and positive | 39.2 | 3.2 |
| Negative blood cultures | 12.3 | 78.2 |
| Hospital mortality, n (%) | 9 (1.1) | 3 (0.9) |

*Other sites of infection included soft tissue infection, bone and ear infection, ISS: Injury Severity Score.
