# Supplementary material for: Enhancer polymorphism rs10865710 associated with traumatic sepsis is a regulator of PPARG gene expression
Source: Crit Care. 2019 Dec 30;23:430. doi: 10.1186/s13054-019-2707-z (PMC6938012; doi:10.1186/s13054-019-2707-z)
Supplement: Supplementary file 3 — Additional file 3: Table S2. Distribution of the rs10865710C/G in the PPARG among trauma patients. [file 13054_2019_2707_MOESM3_ESM.docx]

**Table S2. Distribution of the rs10865710C/G in the PPARG among trauma patients.**

| **Cohort** | **N** | **MAF (%)** | | **Genotypes, n (%)** | | | **HWE** |
| --- | --- | --- | --- | --- | --- | --- | --- |
|  |  | **Patients** | **Databank*** | **Wildtype** | **Heterozygous** | **Variant** |  |
| Internal test | 797 | 33.94 | 34.90 | 350 (43.91) | 353 (44.29) | 94 (11.79) | 0.73 |
| External validation | 334 | 33.98 | 34.90 | 144 (43.11) | 153 (45.81) | 37 (11.08) | 0.70 |

*Data was from HapMap database for Chinese Han Beijings (n=139). MAF: minor allele frequency, HWE: Hardy-Weinberg equilibrium.
